# Primary cells from patients with adult T cell leukemia/lymphoma depend on HTLV-1 Tax expression for NF-κB activation and survival

**DOI:** 10.1038/s41408-023-00841-7

**Published:** 2023-05-03

**Authors:** Rita Hleihel, Hala Skayneh, Hugues de Thé, Olivier Hermine, Ali Bazarbachi

**Affiliations:** 1 Department of Internal Medicine, Faculty of Medicine, American University of Beirut, Beirut, Lebanon; 2 Department of Anatomy, Cell Biology and Physiological Sciences, Faculty of Medicine, American University of Beirut, Beirut, Lebanon; 3 INSERM UMR 944, CNRS UMR 7212, Institut Universitaire d’Hématologie, Université Paris-Cité, Hôpital St. Louis 1, Paris, France; 4 Service d’Hématologie, Assistance Publique, Hôpital St. Louis 1, Paris, France; 5 College de France, PSL research University, Paris, France; 6 Institut Imagine—INSERM U1163, Necker Hospital, University of Paris, Paris, France; 7 Department of Hematology, Necker Hospital, University of Paris, Assistance Publique Hôpitaux de Paris, Paris, France

**Keywords:** Lymphoma, Oncogenes

## Abstract

Adult T cell leukemia/lymphoma (ATL) is an aggressive malignancy secondary to chronic infection with human T cell leukemia virus type 1 (HTLV-1). The viral oncoprotein Tax initiates T cell transformation through activation of critical cellular pathways, including NF-κB. Unexpectedly, Tax protein is not detectable in most ATL cells, in contrast to the HTLV-1 HBZ protein which antagonizes Tax effects. Here, we demonstrate that primary ATL cells from patients with acute or chronic ATL express very low levels of Tax mRNA and protein. Critically, survival of these primary ATL cells is dependent on continued Tax expression. Mechanistically, Tax extinction results in reversal of NF-κB activation, P53/PML activation and apoptosis. Tax drives interleukin-10 (IL-10) expression and recombinant IL-10 rescues the survival of *tax*-depleted primary ATL cells. These results demonstrate the critical role of continued Tax and IL-10 expression for the survival of primary ATL cells, highlighting their relevance as therapeutic targets.

## Introduction

Adult T cell leukemia/lymphoma (ATL) is a rare blood malignancy carrying a dismal prognosis [1], which is secondary to chronic infection with the human T cell leukemia virus type 1 (HTLV-1) [2]. HTLV-1 infects 10–20 million individuals worldwide [3], of which around 5% develop ATL after a long latency period exceeding several decades [3].

ATL development is preceded by oligoclonal expansion of some HTLV-1 infected cells, driven by expression of the viral oncoprotein Tax [4]. Tax is a multifaceted oncoprotein playing pleiotropic functions in ATL leukemogenesis [5–7]. Tax is a transcriptional activator initiating transcription of HTLV-1 mRNAs from the 5’LTR promoter [8]. Tax also alters expression of several cellular genes and interacts with essential signaling pathways imperative for cellular transformation [5, 7–12]. At early stages of HTLV-1 infection, Tax-mediates the constitutive activation of NF-κB, paramount for the proliferation and survival of infected T cells [5, 9]. Tax connects to the IKK kinase complex, resulting in IκBα phosphorylation ubiquitination, and proteasomal degradation, ultimately activating NF-κB, a key regulator of T lymphocytes growth [8–10, 13–16].

Tax oncogenic capacity is well documented, as its sole expression transforms T cells in vitro, and induces leukemia in transgenic mice or flies [17–23]. While the role of Tax in initiating ATL development is well established, its role in maintaining the leukemic phenotype is contentious. Indeed, Tax transcript and protein are not detected in most ATL cells [24–26]. However, primary ATL cells exhibit many properties of Tax-expressing cells, particularly constitutive NF-κB activation [27], proposed to result from mutations targeting the T-cell receptor and the NF-κB pathways [28, 29].

Nevertheless, some indications suggest a role of Tax in the maintenance of the leukemic phenotype in vivo. Transient bursts of Tax expression occur in small fractions of HTLV-1 infected cells or the ATL-derived cell line MT-1 [30, 31]. Anti-Tax antibodies and Tax-specific cytotoxic T lymphocytes were reported in ATL patients [32, 33]. Injection of ATL cells in animals led to the development of Tax-specific CTL [34, 35], while a Tax peptide-pulsed dendritic cell vaccine showed some efficacy in treating Tax-positive ATL patients [36]. Finally, treatment with arsenic trioxide (ATO) and interferon-alpha (IFN), which induce Tax proteasomal degradation, resulted in selective cell death of ATL cells and ensured long-lasting responses in ATL patients [17, 37–42].

HBZ, encoded by the complementary strand of HTLV-1 [43], is constantly expressed in asymptomatic carriers or ATL patients [44]. HBZ decreases Tax expression [45] and inhibits NF-κB activity [46] to counterbalance Tax-induced hyperactivation of NF-κB and preclude senescence [47, 48]. Accordingly, HBZ overexpression in *tax*-transgenic flies rescues Tax-induced cellular transformation [49] providing a direct in vivo evidence for antagonistic roles between Tax and HBZ.

High levels of interleukin-10 (IL-10), an immunosuppressive cytokine modulated by both Tax and HBZ, were reported in ATL patients [38, 50, 51]. IL-10 is an immunosuppressive NF-κB target which enhances the proliferation of HTLV-1-infected cells [52]. Anti-viral therapies of ATL decrease IL-10 levels in mice and patients [38, 42], restoring innate immunity [42].

Here, we demonstrate that the survival of primary ATL cells from patients is dependent on Tax expression and identify IL-10 as a key downstream target. These results strengthen the concept of ATL as Tax-dependent malignancy highlighting its role as a key therapeutic target.

## Methods

### Cell lines and primary ATL cells

HTLV-1 transformed and ATL-derived cell lines (HuT-102, MT-1; gift from K. Ishitsuka)) and HTLV-1-negative cells (CEM, Jurkat) were maintained in RPMI medium supplemented with 10% fetal bovine serum (FBS; Sigma-Aldrich; Germany) and antibiotics.

Blood was collected from two healthy donors, three patients with chronic ATL and three patients with acute ATL after informed consent in accordance with the declaration of Helsinki. This study was approved by the institutional review board of the American University of Beirut. Peripheral blood mononuclear cells (PBMC) were separated using Ficoll and cultured in RPMI supplemented with 10% FBS and antibiotics.

### Short hairpin RNA (shRNA)

Cells were transduced with murine stem cell virus green fluorescent protein (GFP)-lentiviral vectors encoding scrambled (SCR) short hairpin RNA (shRNA) or shRNA against Tax (sh-Tax (1): CAGGCCTTATTTGGACATTTA, sh-Tax (2): CTCAGCTCTACAGTTCCTTAT) or shRNA against HBZ (kindly provided by M. Matsuoka) [53]. Lentiviruses were produced by transient transfection of HEK-293T cells. Infection of HuT-102, MT-1 and primary ATL leukemic cells was performed by spinoculation (3 h at 1500 rpm and 32 °C). GFP-positive transduced cells were sorted 24 h post-spinoculation by flow cytometry (see below). Sorted cells were seeded at the density of 1 million/ml and cell count was performed, using the trypan blue exclusion dye assay, on days 1–7 to assess cell viability following extinction of Tax or HBZ. Experiments were performed once for each shRNA using biological replicates (six ATL patients and two healthy donors) and three times using technical replicates of HTLV-1 infected cells (HuT102 and MT1).

### In situ proximity ligation assays (Duolink®), immunofluorescence and confocal microscopy analysis

Cells were cytospun onto glass slides (5 min, 800 rpm) and fixed with methanol at −20 °C. Protein-protein interactions were visualized using the Duolink® in situ proximity ligation assay (PLA) system (Olink Bioscience; Sweden) [54]. Assays were performed using anti-Tax (168-A51; National Institutes of Health AIDS Research and Reference Reagent Program) and anti-Tax (abcam ab26997; US) primary antibodies, according to the manufacturer’s instructions. Immunofluorescence assays were performed using a chicken polyclonal anti-PML (gift from H. de Thé), or anti-Rel A (Invitrogen; MA5-15160; USA) antibodies. Primary antibodies were revealed by Alexa fluor-488 or 594 labeled secondary antibodies from Abcam. Staining of nuclei was performed using DAPI for 5 min and then coverslips were mounted on slides using a Prolong Anti-fade kit (Invitrogen, P36930; USA). Z-stack Images were acquired using a Zeiss LSM 710 confocal microscope (Zeiss, Oberkochen, Germany). Experiments were performed once using biological replicates (five ATL patients and two healthy donors) and three times using technical replicates of HTLV-1 infected cells (HuT102) and HTLV-1 negative cells (CEM).

### Quantitative PCR

Total RNA was extracted using Trizol (Qiagen cat number 79306). Germany). Experiments were performed starting from 2 µg of RNA in a total of 20 µl. cDNA synthesis was performed using a Revert Aid First cDNA synthesis Kit (Iscript, Biorad; US). SYBR green qRT PCR was performed using the BIORAD CFX96 machine. Primers for Tax (Forward primer: 5’-CGGATACCCAGTCTACGTGT-3’; reverse primer: 5’-GAGCCGATAACGCGTCCATCG-3’), HBZ (Forward primer: 5′-TAAACTTACCTAGACGGCGG-3′; reverse primer: 5′-CTGCCGATCACGATGCGTTT-3′), IL-10 (Forward primer: 5’-GCCTAACATGCTTCGAGATC-3’; reverse primer: 5’-TGATGTCTGGGTCTTGGTTC-3’), IL-1β (Forward primer: 5’- AATTTGAGTCTGCCCAGTTCCC-3’; reverse primer: 5’-AGTCAGTTATATCCTGGCCGCC-3’), Rantes (Forward primer: 5’-ACCACACCCTGCTGCTTTGC-3’; reverse primer: 5’-CCGAACCCATTTCTTCTCTGG-3’), IL-6 (Forward primer: 5’-GGAGACTTGCCTGGTGAA-3’; reverse primer: 5’-GCATTTGTGGTTGGGTCA-3’), and IL-8 (forward primer: 5’- ATGACTTCCAAGCTGGCCG-3’; reverse primer: 5’-GCTGCAGAAATCAGGAAGGC-3’) were used. Individual reactions were prepared with 0.25 µM of each primer, 150 ng of cDNA and SYBR Green PCR Master Mix to a final volume of 10 µl. PCR reaction consisted of a DNA denaturation step at 95 °C for 3 min, followed by 40 cycles (denaturation at 95 °C for 15 s, annealing at 57 °C for 60 s, extension at 72 °C for 30 s). For each experiment, reactions were performed in duplicates and expression of individual genes was normalized to the housekeeping gene Glyceraldehyde-3-Phosphate dehydrogenase GAPDH (Forward primer: 5’-AACTTTCCCGCCTCTCAGC-3′; reverse primer: 5′-CAGGAGGACTTTGGG AACGA-3′). The transcript expression level was calculated according to the Livak method. Experiments were performed once using biological replicates (six ATL patients) and three times using technical replicates of HTLV-1 infected cells (HuT102 and MT1) and HTLV-1 negative cells (Jurkat).

### Flow cytometry and cell sorting

All flow cytometry sorting experiments were performed using a BD FACSAria cell sorter and analyzed by BD FACSDIVA™ software. Patients primary ATL cells, HuT-102 or CEM cell lines were stained with anti CD25-FITC (BD Biosciences, 2 ug/ml; US) before sorting. Sorted cells were analyzed by real-time PCR for Tax and HBZ expression. When indicated, sorted GFP-positive cells were treated with recombinant IL-10 (1 ng/ml) (Biolegend, #571006, US) and cell proliferation was assessed.

Twenty-four hours following transduction of cell lines (HuT-102, MT-1) or primary ATL cells with GFP-lentiviral vectors encoding scrambled (SCR), shRNA Tax or shRNA HBZ, cells were sorted based on GFP expression. The proliferation of GFP^+^ or GFP^−^ cells was assessed using the trypan blue dye exclusion assay. The expression of Tax, HBZ and IL-10 transcripts was assessed by real-time PCR. The expression of Tax protein was assessed by western blot. Cellular localization of RelA (Cell signaling; #D14E12; USA) and PML (homemade, gift from H. de Thé) in these cells was assessed by immunofluorescence assay and confocal microscopy analysis. Finally, protein expression of Dec-1 (Novus biologicals, #NB100-10200; USA), PML (homemade, gift from H.de The), p53 (Santa cruz; #DO-1; Germany), P-p53 (cell signaling #9284; USA) and P-IκBα (Invitrogen, #MA5-15224 US) was assessed by western blot.

### Immunoblot analysis

Cells were washed with PBS and lysed directly by Laemmli buffer. One hundred µg of proteins were loaded onto a 12% SDS-polyacrylamide gel, subjected to electrophoresis, and transferred onto nitrocellulose membranes. Blots were incubated with specific antibodies against Tax (168-A51; National Institutes of Health AIDS Research and Reference Reagent Program), p53 (santa cruz #DO-1; Germany), P-p53 ser15 (cell signaling #9284; USA), Dec-1 (Novus biologicals, NBP227151; USA), P-IκBα (Invitrogen MA5-15224; US), actin (Sigma-Aldrich #A2066; Germany) and GAPDH (Abnova #MAB5476; Taiwan). Bands were visualized by autoradiography, following incubation with luminol chemiluminescent substrate (Biorad Clarity Max ECL Substrate # 1705062; US). Experiments were performed twice using technical replicates of patient C1 and twice using technical replicates of HTLV-1 infected cells (MT1).

### Annexin V assay

The annexin V–fluorescein isothiocyanate kit (Roche) was used to assess apoptosis. The adopted protocol was performed according to the manufacturer’s recommendations. Approximately 5000 cells per sample were analyzed on a Guava flow cytometer (Merk Millipore, Darmstadt, Germany). Experiments were performed once using biological replicates of patients C1 and A1 and three times using technical replicates of HTLV-1 infected cells (HuT102 and MT1).

### Statistical analysis

The *t*-test (two-samples assuming unequal variances) was performed to validate significance: *, ** and *** indicate *p* values ≤ 0.05; 0.01 and 0.001, respectively; *p* values <0.05 were considered significant.

## Results

### Primary cells from ATL patients express Tax

**Fig. 1:**
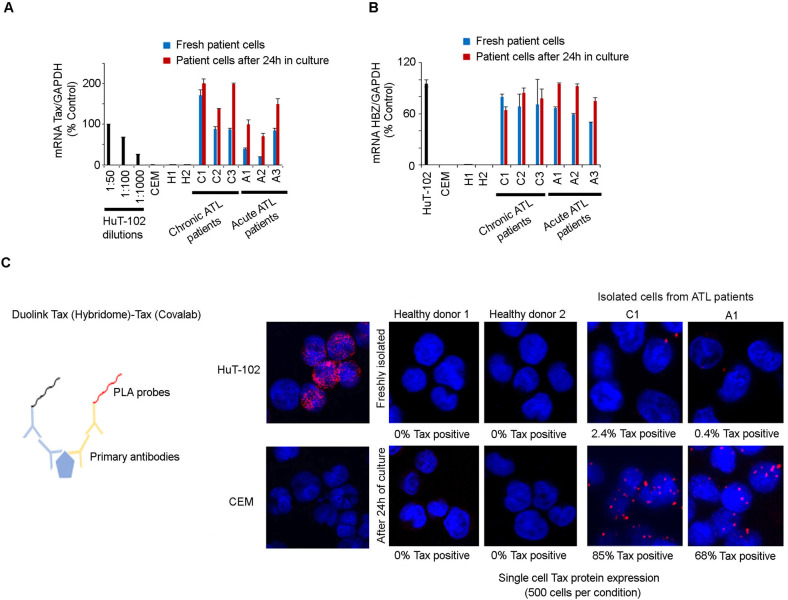
Primary leukemic cells from ATL patients express low levels of Tax mRNA and protein. **A** Transcript levels of Tax in serial dilutions of HuT-102 cDNA in CEM cDNA (black histograms), in freshly isolated (Blue histograms) or short-term (24 h) cultured (Red histograms) primary ATL cells derived from three patients with chronic ATL (C1 to C3), three patients with acute ATL (A1 to A3), and in PBMCs-derived from healthy donors (H1 and H2). Fifty-fold-diluted HuT-102 cDNA in CEM cDNA was taken as control. **B** Transcript levels of HBZ in HuT-102, CEM (black histograms), freshly isolated (Blue histograms) or short-term (24 h) cultured (Red histograms) primary ATL cells derived three patients with chronic ATL, three patients with acute ATL, and PBMCs-derived from healthy donors. Undiluted HuT-102 was taken as control. **C** Duolink® in situ proximity ligation assay, using two different anti-Tax antibodies, performed in HuT-102, CEM, freshly isolated or short-term (24 h) cultured primary ATL cells (chronic ATL C1; acute ATL A1) and PBMCs-derived from two healthy donors. Quantification of Tax protein positive cells was performed on a total of 500 cells per patient.

We explored *Tax* expression in primary cells from three patients with chronic ATL (C1, C2 and C3) and three patients with acute ATL (A1, A2 and A3), using the Tax-expressing cell line HuT-102 as a positive control. Comparing Tax transcript levels in sorted CD25^+^ cells from PBMC of ATL patients, revealed that Tax transcripts in these six patients were 50 to 1000 times less abundant than in HuT-102 cells (Fig. 1A and Supplementary Fig. 1A). Contrarywise, HBZ levels in primary cells from the six tested ATL patients were comparable to those of HuT-102 cells (Fig. 1B and Supplementary Fig. 1B). We also examined Tax expression in these primary leukemic cells after 24 h of ex vivo culture. Slightly higher Tax transcripts levels were detected as compared to freshly isolated samples (Fig. 1A). However, we failed to detect Tax protein neither by western blot (data not shown), nor by immunofluorescence (Supplementary Fig. 1C). In contrast, HBZ transcript levels remained expressed after 24 h of ex vivo culture and comparable to those of freshly isolated cells (Fig. 1B).

We then analyzed Tax protein expression using the highly sensitive in situ proximity ligation assay Duolink®. When using a single primary antibody, Tax protein was detected 24 h after ex vivo culture (but not in uncultured cells) in 40 to 80% of PBMC of all tested ATL patients (Supplementary Fig. 1D), but not HTLV-1-negative CEM cells nor PBMC from HTLV-1 negative healthy donors (Supplementary Fig. 1D). Similarly, using two distinct anti-Tax primary antibodies, Tax protein was again detectable in 68% and 85% of PBMC from the two ATL patients tested (Fig. 1C) but barely detected in uncultured cells (Fig. 1C). These results are similar to those described in PBMC from HTLV-1 healthy carriers [55] suggesting that primary leukemic cells from ATL patients express Tax protein upon short-term ex vivo culture.

### Primary cells from ATL patients depend on Tax expression for their survival

**Fig. 2:**
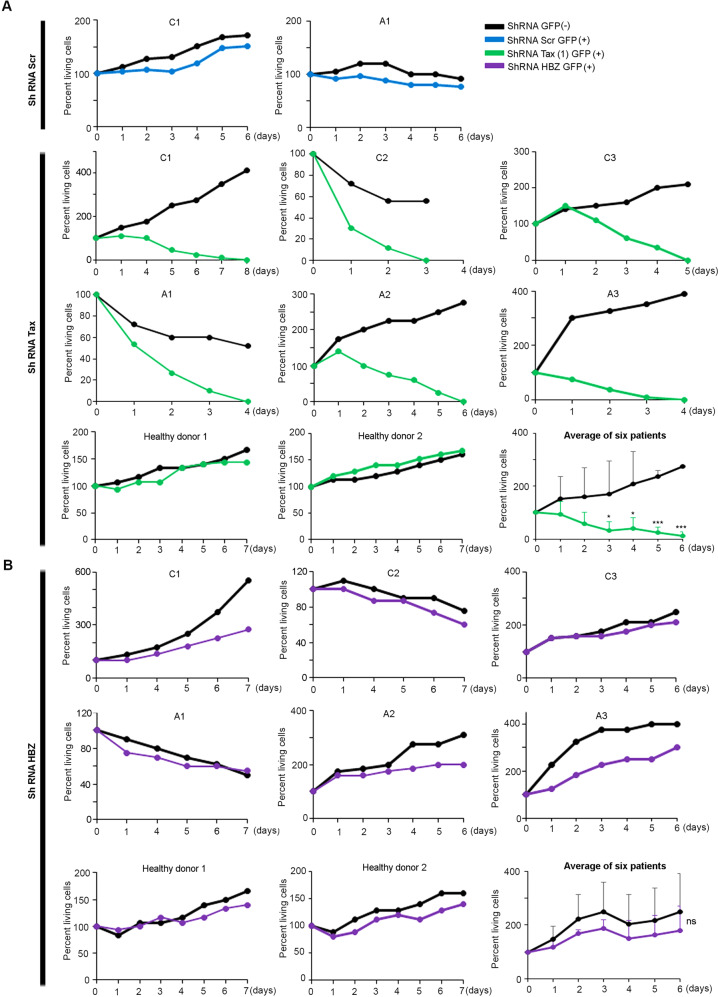
Survival of primary leukemic cells from ATL patients is dependent on Tax expression. **A** PBMCs derived from three patients with chronic ATL (C1 to C3), three patients with acute ATL (A1 to A3), or from two healthy donors were transduced using GFP-lentiviral vectors encoding scrambled (SCR) shRNA or shRNA against Tax. Growth of transduced GFP^+^ or un-transduced GFP^−^ sorted cells was assessed by cell count using the trypan blue exclusion dye assay for up to 7 days after sorting. **B** PBMCs derived from three patients with chronic ATL, three patients with acute ATL, and two healthy donors were transduced using a GFP-lentiviral vector encoding an shRNA against HBZ (shRNA HBZ). Cell growth of transduced GFP^+^ or un-transduced GFP^−^ sorted cells was assessed by cell count using the trypan blue exclusion dye assay for up to 7 days after sorting. The *t*-test was performed on the average of the patients with ATL: *, ** and *** indicate *p* values ≤ 0.05; 0.01 and 0.001, respectively; ns non significant.

To check the dependency of primary ATL leukemic cells on Tax or HBZ expression, we transduced primary leukemic cells from 6 patients with ATL with 2 lentiviral constructs expressing shRNAs specifically targeting Tax (ShTax (1) and ShTax (2)), one at a time, or shRNA specifically targeting HBZ, or scrambled controls. Efficient downregulation of Tax or HBZ expression in lentiviral-infected primary leukemic cells or cell lines was demonstrated by quantitative real-time PCR (Supplementary Fig. 2A, B). Strikingly, downregulation of Tax expression resulted in cell death of primary leukemic cells from all six tested ATL patients (Fig. 2A and Supplementary Fig. 2C), as in HTLV-1-positive cell lines (HuT-102 and MT-1) (Supplementary Fig. 2D). Non-targeting (scrambled) (as well as HBZ shRNA, see below) were ineffective, and GFP-negative uninfected cells were unaffected (Fig. 2A, B and Supplementary Fig. 2E), while expression of Tax sh RNAs did not affect cell growth of 2 HTLV-1 negative healthy donors (Fig. 2A), all excluding off-target effects. Extinction of HBZ expression only slightly affected cell growth of primary leukemic cells from ATL patients (Fig. 2B). A major increase in Tax transcripts was observed upon knock down of HBZ in primary ATL cells (Supplementary Fig. 3A), as reported in cell lines, while Tax knock-down did not affect HBZ expression (not shown). Following HBZ knock down, Tax protein became detectable by Western blot in primary ATL cells (Supplementary Fig. 3B). Hence, HBZ downregulates the expression of Tax protein in primary ATL cells. Collectively, these results directly implicate Tax in the survival of primary leukemic cells.

### Basal NF-κB activation in primary ATL cells is Tax dependent

**Fig. 3:**
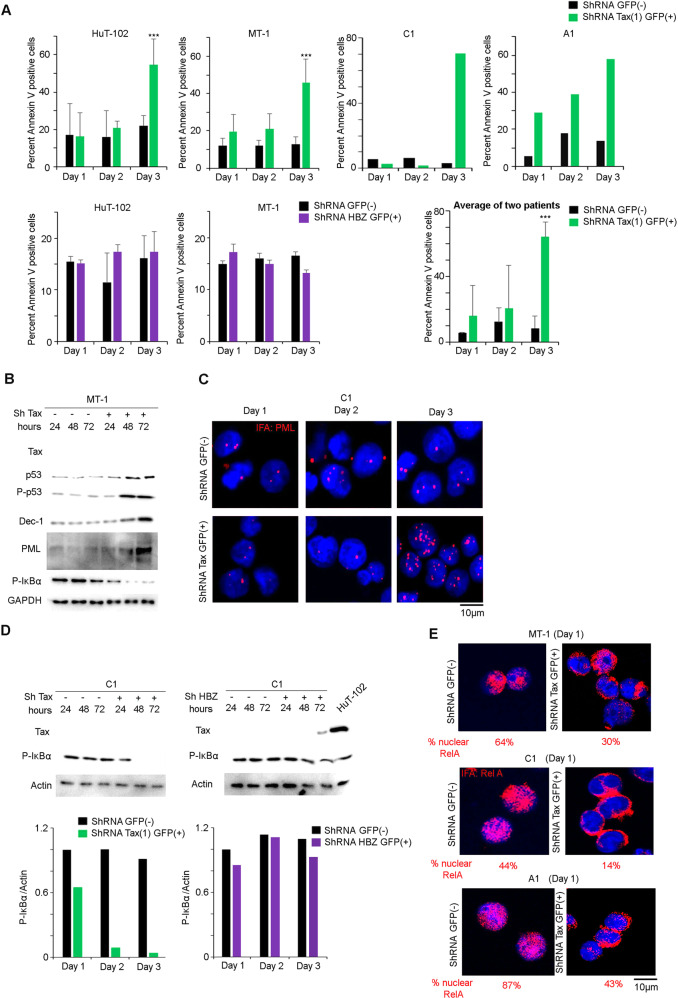
Tax drives NF-kB activation in primary ATL cells. **A** HuT-102 and MT-1 cells and PBMCs derived from one patient with chronic ATL (C1) and one patient with acute ATL (A1), were transduced with shRNA Tax (shTax) or shRNA HBZ (shHBZ). Transduced GFP^+^ or un-transduced GFP^−^ sorted cells were collected after 24, 48 and 72 h post-sorting and were analyzed by Annexin V staining. Histograms represent the percentage of Annexin V positive cells. *, ** and *** indicate *p* values ≤ 0.05; 0.01 and 0.001, respectively. **B** ATL-derived MT1 cells were transduced with shRNA Tax. Transduced GFP^+^ or un-transduced GFP^−^ sorted cells were collected at 24, 48 and 72 h post sorting and were analyzed by western blot using antibodies against Tax, p53, P-p53, Dec-1, PML and P-IκBα. **C** PBMCs derived from one patient with chronic ATL (C1) were transduced with shRNA Tax (shTax). Transduced GFP^+^ or un-transduced GFP^−^ sorted cells were analyzed by immunofluorescence for PML expression. **D** PBMCs derived from one patient with ATL (C1) were transduced using shRNA Tax (shTax) or shRNA HBZ (shHBZ). Transduced GFP^+^ or un-transduced GFP^−^ sorted cells were collected after 24, 48 and 72 h post-sorting and were analyzed by western blot using antibodies against Tax and P-IκBα. Histograms represent densitometry analysis of P-IκBα over actin. **E** ATL-derived MT1 cells or PBMCs derived from one patient with chronic ATL (C1) and one patient with acute ATL (A1) were transduced with shRNA Tax. Transduced GFP^+^ or un-transduced GFP^−^ sorted cells were collected after 24 h post-sorting and were analyzed by immunofluorescence microscopy using anti RelA. The percentage of nuclear RelA in 50 counted cells is indicated.

**Fig. 4:**
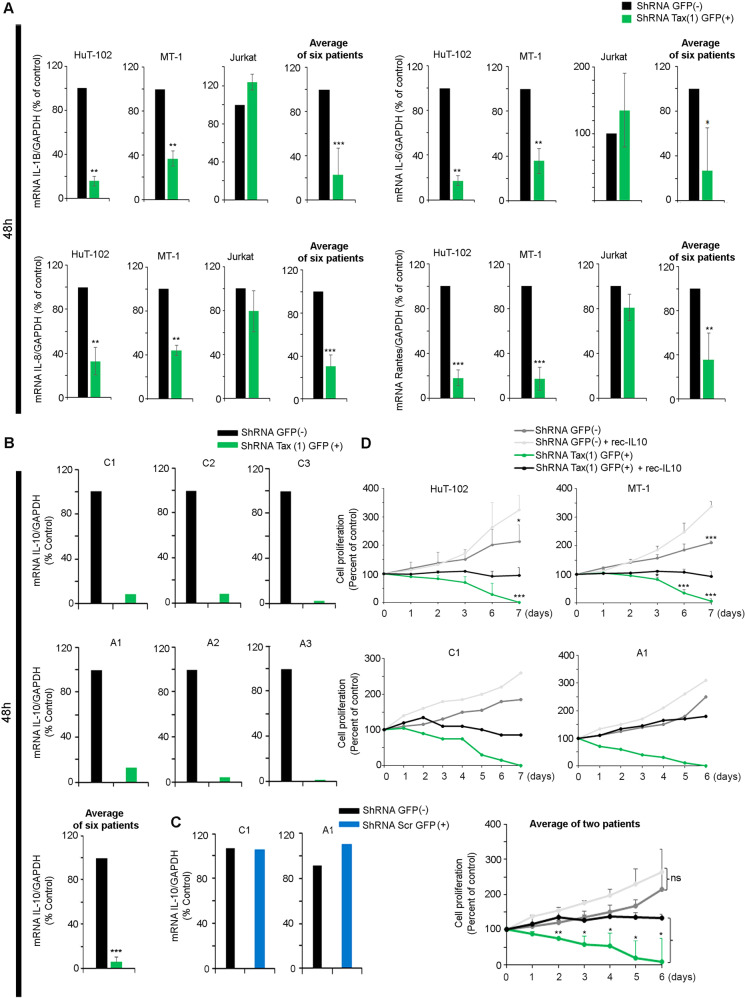
Tax drives IL-10 production in primary ATL cells. **A** Transcript level of IL-1β, IL-6, IL-8 and Rantes in GFP+ sorted cells from HuT102, MT-1, Jurkat and PBMCs derived from six patients with ATL following transduction with shRNA Tax. *, ** and *** indicate *p* values ≤ 0.05; 0.01 and 0.001, respectively. Transcript level of IL-10 in GFP^+^ sorted cells from PBMCs derived from six patients with ATL following transduction with shRNA Tax (**B**; green histograms) or shScr (**C**; blue histograms). The *t*-test was performed on the average of the patients with ATL: *, ** and *** indicate *p* values ≤0.05; 0.01 and 0.001, respectively. **D** HuT-102 cells, MT-1 cells, or PBMCs derived from one patient with chronic ATL (C1), or one patient with acute ATL (A1), were transduced with shRNA Tax. Cell growth of transduced GFP^−^ and GFP^+^ sorted cells was assessed using the trypan blue exclusion dye assay for up to 7 days post-sorting, in the absence or presence of 1 ng/ml of recombinant-IL-10. It is noteworthy that IL-10 levels in the supernatant of PBMCs from ATL patients are around 0.4 ng/ml. *, ** and *** indicate *p* values ≤ 0.05; 0.01 and 0.001, respectively.

A massive increase of apoptosis was observed 3 days post-Tax silencing in MT1 cells, which do not express detectable Tax protein in the basal state, in HuT-102 cells which express high Tax levels, and in primary leukemic cells from two tested ATL patients (Fig. 3A). Induction of apoptosis, was accompanied by a gradual increase in p53 protein and its phosphorylated form P-p53 (Fig. 3B). Tax silencing in MT1 cells also resulted in the upregulation of Dec-1, and increase of PML protein levels, both suggestive for senescence induction (Fig. 3B). Accordingly, a significant induction of PML nuclear body formation was observed 3 days after Tax silencing in primary leukemic cells from one tested ATL patient (Fig. 3C). We also tested the effect of Tax silencing on NF-κB activation. Shutoff of Tax in MT-1 cells (Fig. 3B), as well as in primary leukemic cells from one tested ATL patient (Fig. 3D), resulted in early downregulation of IκB-α phosphorylation. Tax silencing in MT1 cells or primary leukemic ATL cells also resulted in rapid cytoplasmic translocation of RelA, indicative of inactivation of the NF-κB pathway (Fig. 3E). Accordingly, Tax silencing significantly downregulated the expression of the NF-κB target genes interleukin-1 beta, interleukin-6, interleukin-8 and Rantes in Tax-expressing cells (Fig. 4A). In contrast, shutoff of HBZ in MT1 cells (Supplementary Fig. 3C) and in one tested patient with ATL did not affect IκB-α phosphorylation (Fig. 3D) nor the expression of the above mentioned NF-κB target genes (data not shown). Collectively, our results demonstrate that shutoff of Tax in primary ATL cells rapidly abrogates NF-κB activation, followed by activation of P53 and induction of apoptosis.

### IL-10 production in primary ATL cells is Tax dependent

IL10, a key cytokine acting downstream of Tax and NF-κB, plays a critical role in ATL leukemogenesis. Extinction of Tax expression resulted in dramatic downregulation of IL-10 transcript levels in primary leukemic cells from ATL patients (Fig. 4B and Supplementary Fig. 3D) or cell lines (HuT-102 and MT-1) (Supplementary Fig. 3E). Critically, treatment of primary ATL cells or cell lines with physiological levels (1 ng/ml) of recombinant IL-10 after Tax knock-down, rescues their survival (Fig. 4D). These results demonstrate that IL10 production in ATL cells requires the presence of Tax and that IL-10 can overcome the detrimental effects of Tax silencing on ATL cell survival.

## Discussion

In this manuscript we present evidence that primary cells from patients with acute or chronic ATL express very low levels of Tax protein and depend on Tax expression for NF-κB activation, IL-10 production and survival. These results counter the prevalent notion that Tax expression is shutoff at the leukemic stage of the disease.

HTLV-1, the prototypical human oncoretrovirus, initiates the development of ATL [5–7]. Tax, a powerful viral trans-activator, plays a key role in the initiation of ATL [5–7]. Tax drives the proliferation of infected T cells through the constitutive activation of the NF-κB pathway [5, 9]. Tax expression also induces the production of IL-10 [38, 50, 51], which protects ATL cells from innate or adaptive immunity [42]. Yet, Tax was not detected in full blown ATL cells [24–26]. Accordingly, the role of Tax in maintenance of the disease has remained highly controversial. The most prevalent dogma infers the maintenance of ATL phenotype to the accumulation of subsequent somatic mutations [29], and the expression of the HTLV-I antisense protein HBZ whose expression, contrary to that of Tax, is detected in almost all ATL primary cells [44].

The loss of Tax expression in ATL cells was attributed to multiple DNA methylations identified at HTLV-1 5’LTR promoter or even deletion of the 5’LTR [9], the strong immunogenicity of Tax protein ultimately leading to the rapid elimination of Tax-expressing cells by the immune system [56–58], and Tax-induced NF-κB hyperactivation which may result in cellular senescence [47, 48, 59]. Using a highly sensitive technique, in situ proximity ligation assay, we could detect very low levels of Tax protein expression in primary leukemic cells from acute and chronic ATL patients, after a short-term culture. These results indicate that most ATL cells retain the ability to express Tax protein and that the lack of detection of Tax expression in primary ATL cells, particularly at the protein level, is attributed to the lack of sensitivity of the techniques used in previous studies [24, 29, 60].

We show that, only a small percentage of uncultured primary ATL cells barely express very low levels of Tax protein. Upon short-term culture, most ATL cells express Tax protein, albeit also at very low levels. These results are in agreement with previous reports in HTLV-1 asymptomatic carriers, showing that the HTLV-1 sense-strand transcription, encoding Tax and viral structural proteins, is usually silent at a given time in each cell, but gets reactivated upon cellular stress, enabling the transmission of the virus to a new host cell [61]. Indeed, antisense-strand transcription encoding HBZ is stable throughout the stress response. In contrast, the HTLV-1 sense-strand reactivation is highly heterogeneous and occurs in short, self-terminating bursts [61]. Similarly, in the HTLV-1 transformed cell line MT1, latent HTLV-1 provirus reactivates sporadically and transiently in short bursts occurring in a small number of cells at a time [31]. This transient Tax expression stimulates the expression of cellular factors to sustain the survival of neighboring cells [31]. Here, we show that the survival of primary ATL cells depends on this very low level of Tax expression. Importantly, neither the somatic mutations nor HBZ were able to sustain primary ATL cell survival in the absence of Tax. Despite the continuous HBZ expression in all ATL cells, shut down of HBZ had only minimal effects on the survival of primary ATL cells. Yet, knock down of HBZ in primary ATL cells resulted in a major increase in Tax transcripts and protein, allowing the detection of Tax by Western blot. Hence, the continuous expression of HBZ significantly contributes to the downregulation of Tax protein expression in primary ATL cells.

Reversal of Tax-induced NF-κB activation likely contributes to inhibition of proliferation, since NF-κB inhibitors induced apoptosis and selectively decreased HTLV-1-infected cells in the peripheral blood of virus carriers [62]. That Tax shut down reversed NF-κB activation indicates that constitutive NF-κB activation in primary ATL cells [27] is Tax-dependent. Interestingly, somatic mutations, even those targeting the T-cell receptor and the NF-κB pathways [28, 29] were not able to sustain this activation in the absence of Tax. Conversely, HBZ shut down had no effect on NF-κB activation.

ATL patients exhibit high levels of IL-10, that promote proliferation of HTLV-1-infected cells through STAT3 and IRF4 [52], which contribute to ATL maintenance [63]. Here we show that shutoff of Tax in primary leukemic cells from ATL patients, sharply decreases IL-10 levels. Conversely, recombinant IL-10 rescues the ATL phenotype and overcomes the detrimental effects of Tax silencing. These observations suggest that IL-10 is a key effector downstream of Tax, stressing the role of IL-10 as a therapeutic target to enhance the potency of anti-Tax therapies.

In conclusion, our results pinpoint that ATL is not only a virus-initiated leukemia, but also a virus-addicted malignancy, providing a strong rationale for the clinical development of Tax- targeting therapy [64]. Potential candidates include IFN and ATO which induce Tax degradation by the proteasome [17, 37–42], anti-Tax immunotherapy [33] including therapeutic vaccines [36], allogeneic stem cell transplantation through Tax-specific CTL [65], or chimeric antigen receptor (CAT) T or natural killer cells.

## Supplementary information

Reproducibility checklist

Supplemental material

## Data Availability

For original data, please contact bazarbac@aub.edu.lb.
