# Supplementary material for: Primary cells from patients with adult T cell leukemia/lymphoma depend on HTLV-1 Tax expression for NF-κB activation and survival
Source: Blood Cancer J. 2023 May 3;13(1):67. doi: 10.1038/s41408-023-00841-7 (PMC10156663; doi:10.1038/s41408-023-00841-7)

SUPPLEMENTARY LEGENDS

Supplementary Figure 1: (A) Serial dilutions of mRNA from the Tax expressing HuT-102 cell line with mRNA from the HTLV-I negative CEM cell line (black histograms) were performed. Freshly isolated peripheral blood mononuclear cells (PBMC) from HTLV-I negative healthy donors H1 and H2, $CD25^{-}$ (blue histograms) or $CD25^{+}$ (red histograms) sorted cells from three patients with chronic ATL (C1 to C3) and three patients with acute ATL (A1 to A3) were extracted. Transcript levels of Tax were assessed by real-time PCR and 50-fold-diluted HuT-102 cDNA in CEM cDNA was taken as control. (B) Transcript levels of HBZ were assessed by RT-PCR in HuT-102, CEM, healthy donors H1 and H2, $CD25^{-}$ (blue histograms) or $CD25^{+}$ (red histograms) sorted cells from freshly isolated PBMC from three patients with chronic ATL and three patients with acute ATL. HuT-102 transcripts were taken as control. (C) Confocal microscopy of Tax protein expression by immunofluorescence in HuT-102, CEM and primary leukemia cells derived from ATL patients. (D) Duolink® *in situ* proximity ligation assay, using one anti-Tax antibody, performed in HuT-102, CEM, freshly isolated or short-term (24h) cultured primary ATL cells and PBMCs-derived from two healthy donors. Quantification of Tax protein positive cells was performed on a total of 500 cells per patient.

Supplementary Figure 2: (A) Transcript level of Tax in GFP^{+} sorted cells from PBMCs derived from 3 patients with chronic ATL (C1 to C3), 3 patients with acute ATL (A1 to A3), HuT-102 and MT-1 cell lines following transduction with shScr or shRNA Tax (1 or 2). *, ** and *** indicate p values ≤ 0.05; 0.01 and 0.001, respectively (B) Transcript level of HBZ in GFP^{+} sorted cells from PBMCs derived from 3 patients with

chronic ATL, 3 patients with acute ATL, HuT-102 and MT-1 cell lines following transduction with shHBZ. *, ** and *** indicate p values ≤ 0.05; 0.01 and 0.001, respectively (C) PBMCs derived from one patient with chronic ATL (C1) or one patient with acute ATL (A1) were transduced using GFP-lentiviral vectors encoding a second shRNA against Tax. Growth of transduced GFP^+ or un-transduced GFP^- sorted cells was assessed by cell count using the trypan blue exclusion dye assay for up to 7 days after sorting. (D-E) Cell growth of transduced GFP^+ or un-transduced GFP^- sorted HuT-102 and MT1 with shRNA Tax (D) or shRNA HBZ (E). Cell growth was assessed by cell count using the trypan blue exclusion dye assay for up to 7 days after sorting. *, ** and *** indicate p values ≤ 0.05; 0.01 and 0.001, respectively; ns=non significant.

Supplementary Figure 3: (A) Transcript level of Tax in GFP^+ sorted cells from PBMCs derived from 3 patients with chronic ATL (C1 to C3) and 3 patients with acute ATL (A1 to A3) following transduction with GFP-lentiviral vectors encoding shRNA against HBZ (shRNA HBZ). (B) Transduced GFP+ or un-transduced GFP− sorted PBMCs derived from one patient with chronic ATL (C1) with shRNA HBZ were collected on days 1, 2 and 3 post sorting and were analyzed by western blot for Tax expression. Extracts from undiluted HuT-102, or diluted in CEM cells at 10 to 100 -folds were used as controls. (C) ATL-derived MT1 cells which do not express Tax at the protein level, were transduced using GFP lentiviral vectors encoding shRNA HBZ. Transduced GFP^+ or un-transduced GFP^- sorted cells were collected at 24h, 48h and 72h post-sorting and were analyzed by western blot using antibodies against Tax and p-IκB-α. (D) Transcript level of IL-10 in GFP^+ sorted cells from PBMCs derived from 1 patient with chronic ATL (C1) and 1 patient with acute ATL (A1) following transduction with a second shRNA Tax. (E) Transcript level of IL-10 in GFP^+ sorted HuT-102 and

MT-1 cell lines following transduction with shRNA Tax. *, ** and *** indicate p values ≤ 0.05; 0.01 and 0.001, respectively.

Supplementary Figure 1

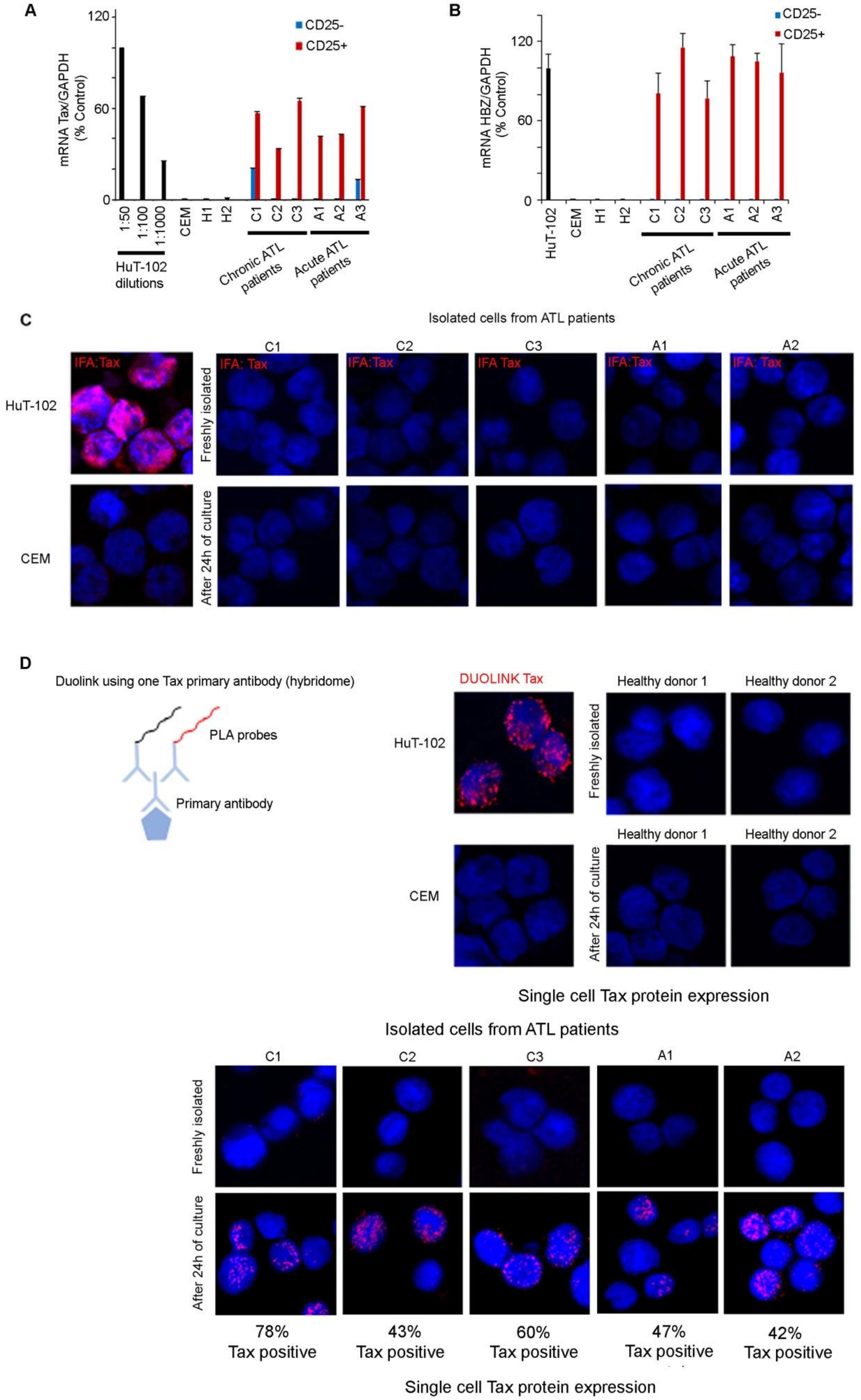

Supplementary Figure 2

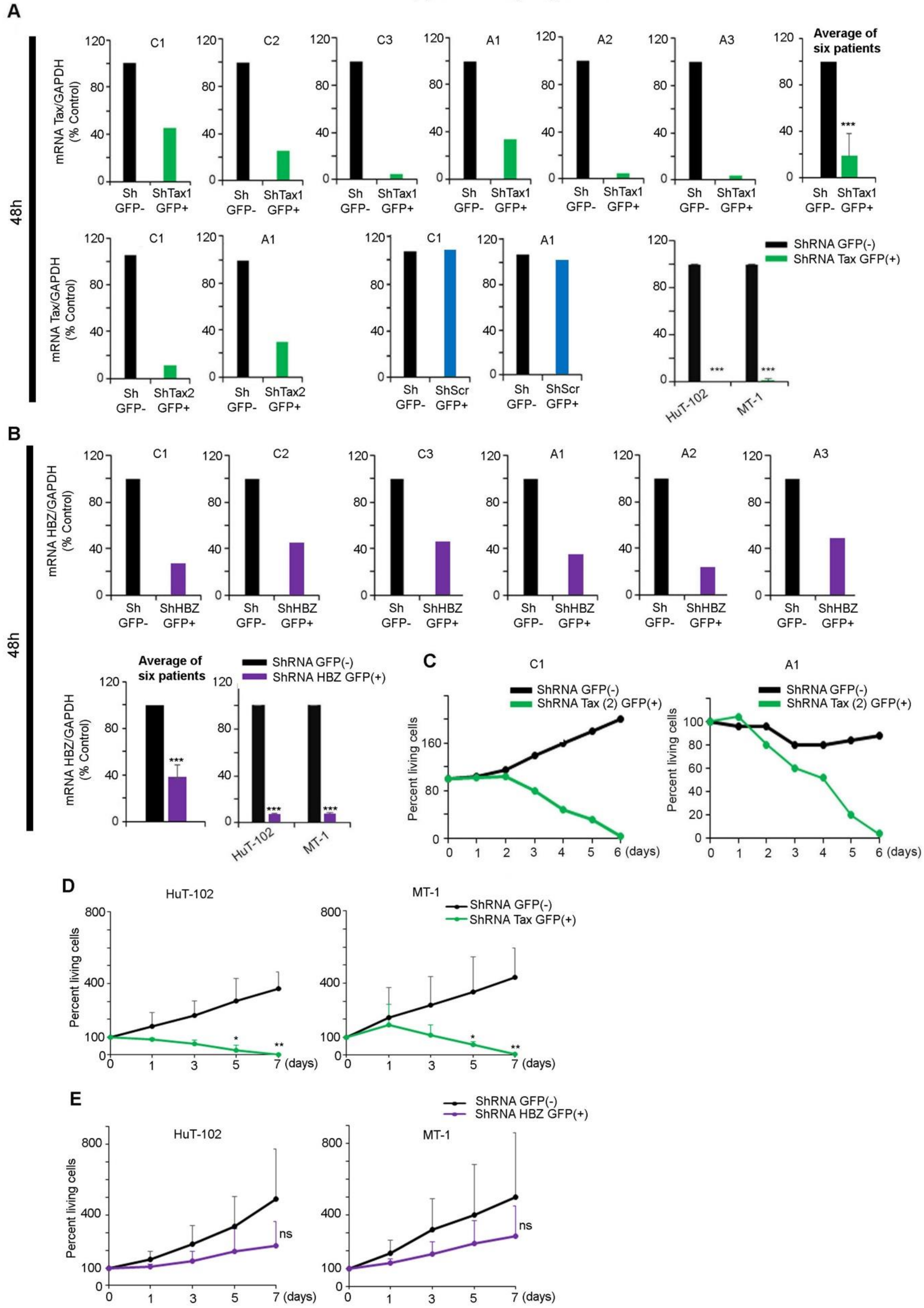

Supplementary Figure 3

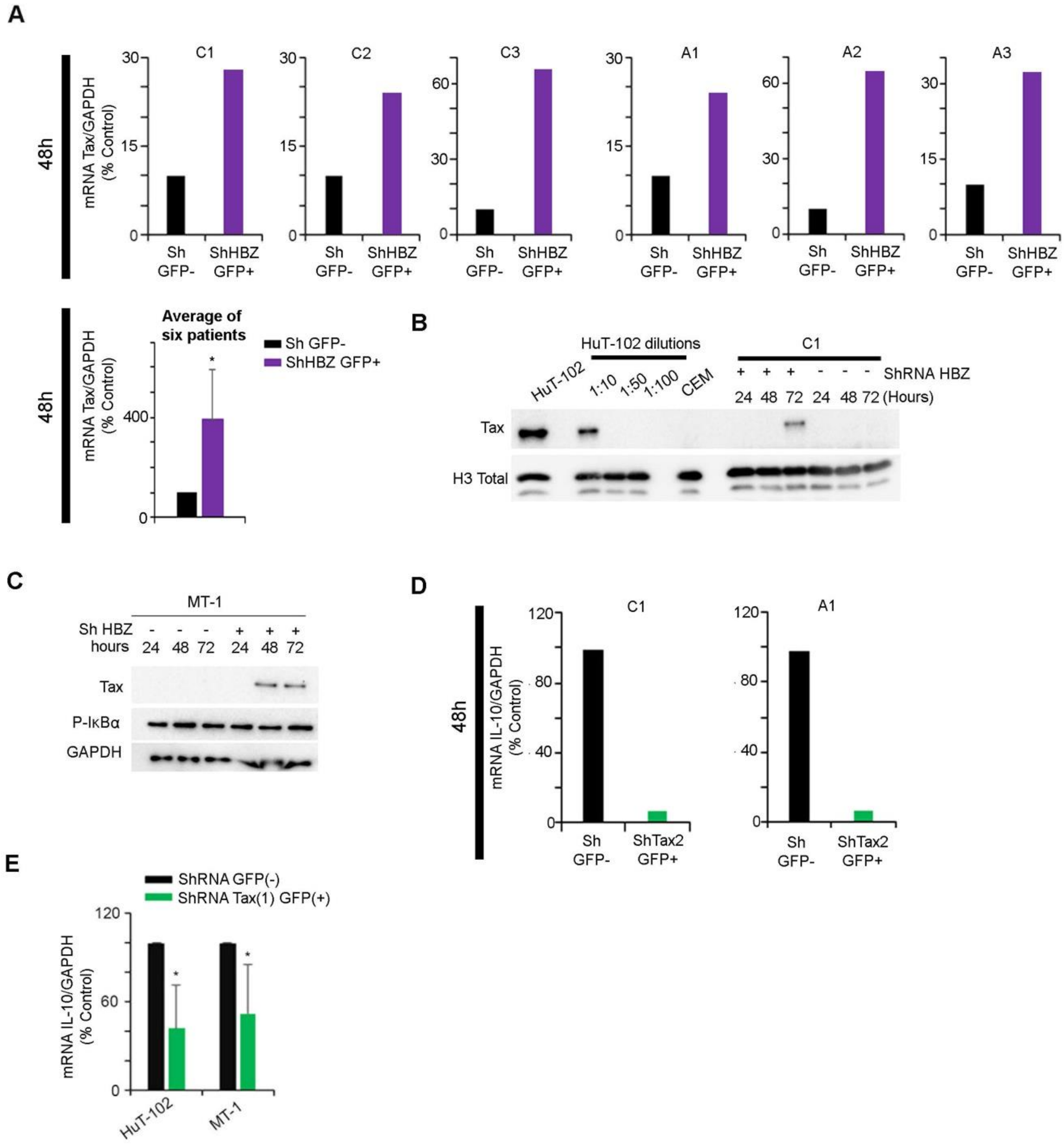

Supplement: Supplementary file 2 — Supplemental material [file 41408_2023_841_MOESM2_ESM.pdf]
